# Jackhammer Esophagus: From Manometric Diagnosis to Clinical Presentation

**DOI:** 10.1155/2019/5036160

**Published:** 2019-03-03

**Authors:** Marianne Clément, Wen Jing Zhu, Elissaveta Neshkova, Mickael Bouin

**Affiliations:** Division of Gastroenterology, Gastro-Intestinal Motility Center, Centre Hospitalier de l'Université de Montréal (CHUM), Montreal, QC, Canada

## Abstract

**Background:**

Jackhammer esophagus is a hypercontractile esophageal disorder recently brought to light with the advent of high resolution manometry (HRM). As little is known about its clinical presentation, the aim of this study was to identify the clinical characteristics associated with this new gastrointestinal motility disorder.

**Methods:**

A retrospective study was conducted on patients visiting the CHUM's Gastro-Intestinal Motility Center from January 2015 to December 2017. The HRM diagnoses were collated in a database along with age and sex of every individual. The latest Chicago classification (version 3.0) was used. Among all the patients subjected to HRM, those diagnosed with Jackhammer esophagus were included in the study. Patient charts were reviewed to collect relevant demographic and clinical data.

**Key Results:**

A total of 36 patients with Jackhammer esophagus were included (62 ± 13 years age, 89% females). Their main symptoms were dysphagia (72%), pyrosis (42%), retrosternal chest pain (36%), and epigastralgia (33%). Other manometric findings were hypertonia (22%) and/or inadequate relaxation (19%) of the lower esophageal sphincter. Among the 26 patients who had esogastroduodenoscopy, hiatal hernia was seen in 3 patients. Pathological gastroesophageal reflux was found in 4 of the 10 patients investigated by pH-monitoring.

**Conclusions and Inferences:**

Jackhammer esophagus represents 3% of the HRM diagnoses in this study, with a significant female preponderance. In more than two-thirds of cases, the clinical presentation of Jackhammer esophagus is dysphagia.

## 1. Introduction

High resolution manometry (HRM) is the gold standard for the diagnosis of esophageal motility disorders [1]. These are defined by the Chicago classification, which was revised in 2014 (version 3.0) [2]. The esophageal motility disorders are achalasia, esophagogastric junction outflow obstruction, major disorder of peristalsis (distal esophageal spasm, Jackhammer esophagus, absent contractility), and minor disorders of peristalsis (ineffective esophageal motility, fragmented peristalsis) [2].

HRM allows evaluation of the esophagogastric junction relaxation through the integrated relaxation pressure (IRP), expressed in mmHg. In the latest Chicago classification, the IRP is the only recognized HRM parameter to make conclusions about the lower esophageal sphincter (LES) relaxation, even though basal and residual LES pressures are also measured [2]. HRM assesses the esophageal contractile vigor through the distal contractile integral (DCI) [2, 3]. This parameter, expressed in mmHg.s.cm, is calculated by combining amplitude, duration, and length of the peristaltic wave [2].

**Figure 1:**
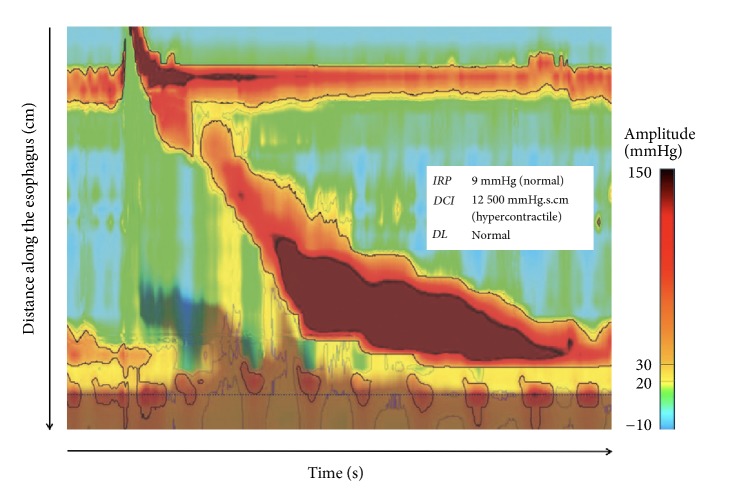
High resolution manometry with pressure topography of a patient with Jackhammer esophagus. This hypercontractile swallow has normal integrated relaxation pressure (IRP) and distal latency (DL), with a distal contractile integral (DCI) superior to 8 000 mmHg.s.cm.

The diagnostic criteria for Jackhammer esophagus (JE) are at least 20% of swallows with a DCI of > 8000 mmHg.s.cm [Figure 1]. In JE, peristalsis is preserved and the distal contractile latency is normal, which differentiates it from achalasia and distal esophageal spasm, respectively [4]. Jackhammer esophagus is a hypercontractile esophageal disorder, described less than a decade ago [4]. JE can be diagnosed even if the LES relaxation is impaired, when the median IRP is above the upper limit of normal range, as long as peristalsis is intact [3].

The pathophysiology of JE remains uncertain, even though some observational studies suggested an association with esophagogastric outflow obstruction [5, 6], gastroesophageal reflux disease (GERD) [2, 5–7], and eosinophilic esophagitis [5]. In a study, JE was observed in one patient with eosinophilic infiltration of the muscularis propria, in conjunction with elevated peripheral IgE levels but normal peripheral blood eosinophil levels. Endoscopic ultrasonography showed circumferential hypertrophy of the muscle layer. This entity has been referred to “eosinophilic esophageal myositis” [8]. Some cases of JE are thought to be idiopathic, in the context of a primary motility disorder [6]. It has been observed that the esophageal muscle thickness was increased on ultrasound in some patients with JE and that the circular and longitudinal layers showed asynchrony [4]. A hypothesis to explain this is an abnormal cholinergic activity within the esophageal muscle innervation. Regardless of the etiology of JE, the extreme hypercontractility is mainly located in the third contractile segment of the esophagus [4].

A small number of observational studies on JE used the latest Chicago classification criteria and thus sparse data are available regarding demographic and clinical characteristics of JE patients [3, 5]. Studies showed that DCI values of > 8000 mmHg.s.cm are rarely encountered in control healthy subjects and are usually associated with symptoms, such as dysphagia and chest pain [3–5, 7].

As there have not been any prospective studies addressing the treatment of JE, its management approach is yet to be validated [2, 7]. Observational studies suggest that possible pharmacological approaches include calcium channel blockers [1, 7], nitrates [1, 7], phosphodiesterase-5 inhibitors [2, 7], anticholinergics [2, 5, 9], and low dose antidepressants [7]. Proton pump inhibitors can be used in patients with JE [7]. Potential endoscopic approaches are botulinum toxin injection [7, 10], pneumatic dilation [7], and peroral endoscopic myotomy (POEM) [7, 11]. In the study mentioned above, where a patient with JE was found to have an eosinophilic infiltration of the muscularis propria, prednisone was used in combination with POEM [8].

Since JE is a manometric diagnosis that was recently introduced and redefined, little is known about its clinical presentation. The need to identify the primary symptoms of JE and if there is a sex predominance is crucial to incite clinicians to request an HRM, when clinical suspicion is high, permitting an earlier diagnosis. An important first step in achieving an understanding of the pathogenesis and obtain a consensus treatment approach is to procure complete clinical information about a cohort of patients, using the latest diagnostic criteria. Thus, the aim of our study was to identify the demographic and clinical characteristics of patients with Jackhammer esophagus.

## 2. Materials and Methods

### 2.1. Study Design and Study Population

This was a retrospective observational study conducted at the* Centre Hospitalier de l'Université de Montréal *(CHUM), Canada. Consecutive HRM studies performed in our tertiary Gastro-Intestinal Motility Center from January 2015 to December 2017 were reviewed. All manometric diagnoses along with age and sex of all patients were compiled in a database. Patients, 18 years of age or older, with the diagnosis of JE according to the current (version 3.0) Chicago classification were included in the study. Diagnostic criteria for JE were the occurrence of at least 20% of contractions with DCI > 8000 mmHg.s.cm, together with intact peristalsis and distal latency. The HRM reports were reviewed by one of the authors (MC), to confirm that if the diagnostic criteria were met for each included patient. Exclusion criteria were history of esophageal cancer and/or past esophagogastric surgery. This study was approved by our institutional research ethics committee.

### 2.2. High Resolution Manometry

All HRM studies were performed by solid state Sandhill HRM system (Diversatek, Milwaukee, Wisconsin, USA). The HRM catheter comprised 36 circumferential sensors distributed at 1 cm intervals. After at least an 8-hour fast, the patients were placed in the supine position with the head of the bed elevated at 30 to 45 degrees. The manometry catheter was inserted transnasally by a trained nurse and positioned to record from the pharynx to the stomach. After calibration and baseline recording, ten 5 mL saline swallows were recorded at thirty seconds intervals. For the calculation of DCI, expressed in mmHg.s.cm, only the pressures greater than 20 mmHg were used by the software. Normal IRP values vary according to HRM manometer model. Normal median IRP for the Sandhill manometer used in our motility center is inferior to 20 mmHg. IRP values of 20 mmHg or higher are elevated and consistent with an esophagogastric junction outflow obstruction [12].

### 2.3. Data Collection

The electronic medical records of all the included patients diagnosed with JE were reviewed to collect clinical data. Clinical variables analysed were age at the time of diagnosis, sex, reported symptoms, comorbidities, medications, history of tobacco use, and alcohol consumption. No routine dysphagia or symptoms questionnaire was performed in every patient undergoing an esophageal HRM. During each HRM study of patients with JE, DCI values for all the 10 swallows performed were assessed. Concomitant manometric measurements collected were basal LES pressure, IRP, and residual LES relaxation pressure. Upper digestive endoscopy, biopsies, pH-metry, and barium swallow results were also compiled in the database. Available information on the treatment course and follow-up was also collected, for each included patient.

### 2.4. Statistical Analysis

Statistical analysis was performed using Microsoft Excel software. Means and respective standard deviations were calculated for continuous variables. Categorical variables were expressed as percentages of frequencies. Additional statistical analyses were performed to compare the JE patients with the patients having the others motility disorders. The Student's t-test was used for the comparison of the mean age in each group and also to compare HRM data between subgroup of JE patients. The* Chi*-square test was used to compare the sex distribution in each group compared to JE and also compare main symptoms between subgroups of JE patients. The p-values below 0.05 were considered statistically significant.

## 3. Results

**Figure 2:**
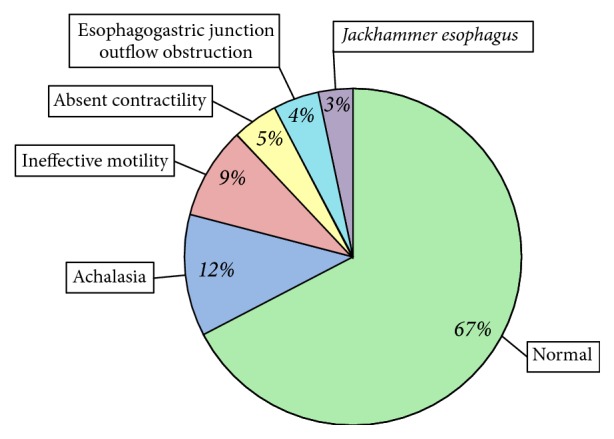
The manometric diagnosis of the 1099 high-resolution manometry studies performed at our tertiary motility center during the study period of three years (January 2015 to December 2017). The percentages indicate the proportion of all patients diagnosed with the associated esophageal motility disorder.

A total of 1099 patients (679 females/420 males) were referred for an esophageal HRM to our Gastro-Intestinal Motility Center during the time period studied. Their mean age was 57 ± 16 years at diagnosis (range 18 to 97 years). We found that 67% of all the HRM were normal, 12% met the criteria for achalasia, 9% were an ineffective esophageal motility, 5% were an absence of contractility, and 4% were an esophagogastric junction outflow obstruction. Jackhammer esophagus was identified in 37 patients, representing 3% of all the manometric diagnoses [Figure 2]. One male patient with JE was excluded of from the study because he had a Nissen fundoplication prior to HRM.

The 36 JE patients included had a mean age of 62 ± 13 years (range 24 to 87 years), and, of these, 89% were females. Comorbidities in these patients included cardiovascular, endocrine, rheumatologic, and pulmonary diseases. Two JE patients had limited systemic scleroderma, also known as CREST syndrome (Calcinosis, Raynaud's phenomenon, esophageal dysmotility, sclerodactyly, telangiectasias). Past or current tobacco use was found in 31% of the JE patients and alcohol use was found in 19%. A total of 21 (58%) patients were on proton pump inhibitors (PPIs) before undergoing HRM [Table 1].

We compared the demographic characteristics of patients diagnosed with JE and other motility disorders diagnosed in our study population [Table 2]. JE patients were older than patients diagnosed with ineffective motility and those having a normal HRM. Mean age was not statistically different between JE and every other HRM diagnosis, including achalasia. The sex distribution of JE patients, with a female preponderance, was statistically different compared to every other motility disorder diagnosed in our study population (p < 0.01).

**Figure 3:**
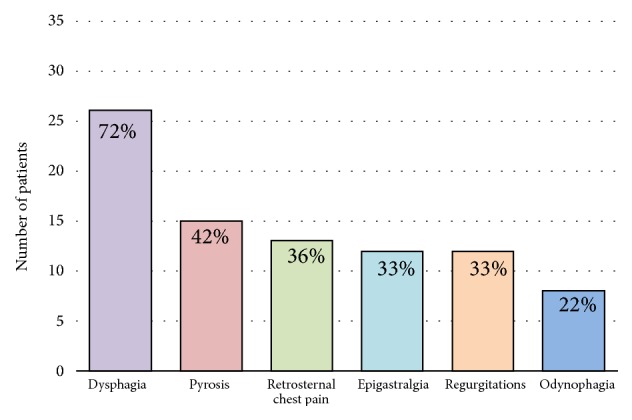
Symptoms of the patients diagnosed with Jackhammer esophagus. The percentages in each column indicate the proportion of patients having the symptom.

Dysphagia was the main symptom in patients with Jackhammer esophagus, affecting 26 (72%) patients [Figure 3]. Other presenting symptoms noticed by the patients with JE were pyrosis (42%), retrosternal chest pain (36%), epigastralgia (33%), regurgitation (33%), and odynophagia (22%).

All JE patients had preserved peristalsis during HRM [Table 3]. More than half of the patients had at least 40% of hypercontractile esophageal contractions during their HRM. The mean DCI of the hypercontractile esophageal contractions was 11 700 mmHg.s.cm, with the highest observed DCI being 47 740 mmHg.s.cm. Other HRM findings were LES hypertonia (more than 45 mmHg) in 22% of cases. Inadequate relaxation of the LES demonstrated by an elevated median IRP (≥ 20 mmHg) was found in 8 (22%) patients. An elevated residual LES pressure (≥ 8 mmHg) was found in 7 (19%) patients. Four of the patients having one or both the findings of abnormal LES relaxation conditions had them concomitantly.

As described above, 8 patients were found to have an esophagogastric junction outflow obstruction, defined by an elevated IRP, being above 20 mmHg. Their mean age was 73 years which was significantly older than patient with a normal IRP who had a mean age of 59 years (p < 0,05). Presenting symptoms did not differ between those two subgroup of JE patients concerning dysphagia (p = 0,25), pyrosis (p = 0,87), and chest pain (p = 0,83). The mean, median, and maximal DCI of JE patient with an elevated IRP were not statistically different from the other JE patients. They also did not have a higher percentage of hypercontractile contractions compared to JE patients having a normal IRP.

Results concerning complementary investigations were assessed for JE patients [Table 4]. Esogastroduodenoscopy results were available for 26 patients and were normal for 18 patients. Abnormal findings during endoscopy included hiatal hernia, longitudinal striae, and impression of esophageal dilatations and of increased LES tone. Among the 13 available biopsy results, 11 were normal. Among the patients with abnormal histology, one had lymphocytic exocytosis and one had esophagitis without eosinophilia. During the study period, 10 patients had a 24 hours' pH-monitoring. Pathological gastroesophageal reflux was demonstrated in 4 patients and hypersensitive esophagus was found in one. Among the 8 patients who had a barium swallow, 4 showed normal results. Three demonstrated spastic contractions of the esophagus and one showed incomplete relaxation of the cricopharyngeal muscle.

Information about treatment was found in the charts of 4 patients. None of the study JE patients had endoscopic treatment. Pharmacologic treatments included addition of a CCB plus a PPI for one patient, increasing the dosage of a PPI for one, adding a PPI for one, and adding citalopram, an antidepressant medication, for the patient with hypersensitive esophagus. All the patients noticed improvement of their symptoms, with a mean follow-up of 11 months (range 2-26).

## 4. Discussion

The highlight of the present study on Jackhammer esophagus is the finding of a significant female preponderance and association with dysphagia. The proportion of patients (3%) having JE diagnosis referred to our tertiary motility center is similar to the 4% proportion reported by a recent study from Texas, USA [5]. The strong female predominance found in the present study appears to be novel as previous studies, with a small number of patients, reported variable results regarding distribution between sexes [3, 5]. The mean age of patients with Jackhammer esophagus was 62 years, in the sixth decade, consistent with past studies, where mean age was 55 and 68 years [3, 5].

We noticed that dysphagia is a consistent predominant symptom of patients diagnosed with JE, as observed in few earlier studies. It was reported before that about 70% of patients with JE presented with dysphagia [5, 11, 13], which is similar to our current finding.

Surprisingly, two of the JE patients in the present study had limited CREST scleroderma. In such rheumatologic disease, a decrease or even an absence of esophageal contractions is normally anticipated. However, the marked esophageal smooth muscle hypercontractility of JE seems to overcome the pathogenesis of esophageal hypomotility usually found in scleroderma, in those two patients.

In our study, a subgroup of JE patients have accompanying esophagogastric junction (EGJ) outflow obstruction, as reported before in previous studies [5, 6]. This suggests a possible role of EGJ obstruction in the pathogenesis of JE. Hypercontractility seen in JE may be “fighting contractions”, within the esophageal body, to overcome an obstruction at the LES level [5]. It has been demonstrated that EGJ obstruction, experimentally or after gastric banding, can induce smooth muscle hypertrophy and hypercontractile contractions in the distal esophagus [4]. This EGJ obstruction may have been the possible cause of esophageal hypercontractility in some of the 8 JE patients (22%) with an elevated IRP. Nevertheless, in our study, there was no significant correlation between DCI and IRP. In other words, the recorded DCI in patient with EGJ outflow obstruction were not higher than the ones recorded in the rest of the JE patients. Our study showed that JE patients with EGJ outflow obstruction were significantly older compared to the other JE patients. This finding might suggest that the same pathophysiologic mechanisms leading to hypercontractile contraction in the distal esophagus could eventually also lead to an impaired relaxation at the esophagogastric junction with advancing age, although this is only a hypothesis.

In fact, the natural course of JE is not well understood. One study reported a possible progression from JE to type III achalasia [3]. The manometric predictor of this progression was an impaired EGJ relaxation (elevated median IRP) at the time of the initial HRM that diagnosed JE [3]. The outflow obstruction is thought to cause motility “after load” against which the esophageal body has to contract more vigorously [3]. This study suggests a possible continuum between JE and type III achalasia in a subgroup of patients [3]. This subgroup of patients could benefit from more aggressive endoscopic treatment at the level of the LES to prevent deterioration of esophageal function [3]. As no JE patient in our study population had a second esophageal HRM performed, such observations about JE's evolution were not possible.

In our study, the minority of JE patients underwent pH-monitoring, but 4 patients out of ten had GERD. Thus, a possible relationship with gastroesophageal reflux disease is noticed in our study, as reported in other series [4, 5]. This suggests that a 24-hour pH-monitoring should be done in the patients with a diagnosis of JE to identify the patients who could potentially benefit from a PPI therapy, if GERD is present. Studies showed that antireflux therapy could alleviate symptoms in patients with JE and was also found to normalize the HRM in a patient in one study [4]. When GERD is present, treatment approach should first focus on the resolution of pathological gastroesophageal reflux with a PPI [2].

Even though a link between JE and eosinophilic esophagitis is suggested in an earlier study [5], we did not find such association in ours. Also, none of our patients was found to have eosinophilic infiltration of the muscularis propria as demonstrated in one study [8]. In fact, no POEM was performed in any and thus no muscle sampling could be done during this procedure, as performed in a later study [8].

Limitations of this study are attributable to its retrospective design. No validated dysphagia questionnaire was routinely used. Symptoms were thus collected using a less reliable method. Also, only a small number of patients were treated and followed up at our tertiary center. This can be explained by the fact that the referring physicians were largely from community hospitals. The undertaken treatments in the majority of JE patients are thus unknown, as we did not have access to their medical files. Thus, unfortunately, minimal information about treatment efficacy was available in this study. A positive aspect of this study was the 3 years' duration of the study, permitting a relatively large number of JE patients to be included for analysis of their demographic characteristics and presenting symptoms.

In summary, this study shows that a significant proportion of HRM diagnoses in our tertiary Gastro-Intestinal Motility Center consisted of Jackhammer esophagus. We noticed a strong female preponderance among JE patients, which is significantly different from the other diagnosed motility disorders. The patients are usually diagnosed in their sixties. The symptoms most frequently reported by JE patients are dysphagia, pyrosis, and chest pain. Even though the precise etiology of JE is unclear, its association with GERD and EGJ outflow obstruction is suggestive of possible role of these conditions. Other cases are probably caused by a primary smooth muscle hypercontractility, whose pathophysiology is yet to be identified, but could be explained by cholinergic modulation [4].

Further research is necessary to better understand the clinical characteristics and the natural history of JE patients. Exploration of the different therapeutic modalities for Jackhammer esophagus is crucial to standardise its management. Prospective randomized trials concerning pharmacologic and endoscopic treatment options are required to accomplish that goal.

## Figures and Tables

**Table 1:** Demographic characteristics and comorbidities of patients with Jackhammer esophagus.

Baseline characteristics	Total n = 36 (%)
*Age (years)*	
Mean age ± standard deviation	62 ± 13
Range	24 - 87

*Sex*	
Females	32 (89)
Males	4 (11)

*Comorbidities*	
Digestive	
GERD	8 (22)
Peptic ulcer disease	4 (11)
Irritable bowel syndrome	2 (5)
Gastroparesis	1 (2)
Primary biliary cholangitis	1 (2)
Cardiovascular	
Dyslipidemia	8 (22)
Hypertension	7 (19)
Coronary artery disease	4 (11)
Atrial fibrillation	3 (8)
Endocrine	
Diabetes	7 (19)
Hypothyroidism	6 (17)
Rheumatologic	
Osteoporosis	4 (11)
Limited scleroderma (CREST)	2 (5)
Fibromyalgia	2 (5)
Rheumatoid arthritis	2 (5)
Polymyositis	1 (2)
Gout	1 (2)
Pulmonary	
COPD	5 (14)
Asthma	4 (11)
Interstitial lung disease	2 (5)
Oncologic	
Breast cancer	3 (8)
Renal cancer	1 (2)

*Lifestyle habits*	
Tobacco (past or current)	11 (31)
Alcohol (past or current)	7 (19)

PPI use prior to HRM	21 (58)

GERD: gastroesophageal reflux disease. CREST: calcinosis, Raynaud phenomenon, esophageal dysmotility, sclerodactyly and telangiectasia. COPD: chronic obstructive pulmonary disease. HRM: high-resolution manometry.

**Table 2:** Comparison of age and sex distribution of the patients with Jackhammer esophagus (JE) and the other motility disorders diagnosed during HRM.

	JE	Normal	Achalasia	Ineffective motility	Absent contractility	EGJ outflow obstruction	Combination of the other HRM diagnosis
*Mean age, years*	62 ± 13	57 ± 15	59 ± 19	54 ± 19	56 ± 18	62 ± 12	57 ± 16
(±standard deviation)							
* p-value*	N/A	0,03	0,32	< 0,01	0,06	0,95	0,05
(compared to JE)							

*Sex distribution*							
Females	89%	64%	40%	62%	60%	65%	62%
Males	11%	36%	60%	38%	40%	35%	38%
* p-value*	N/A	< 0.01	< 0.01	< 0.01	< 0.01	< 0.01	< 0.01
(compared to JE)							

**Table 3:** Manometric characteristics in patients with Jackhammer esophagus.

	Total n = 36 (%)
*DCI values*	
Mean overall DCI (mmHg.s.cm)	8 100
Mean DCI among DCI > 8000 (mmHg.s.cm)	11 700
Range of hypercontractile DCI (mmHg.s.cm)	8 000 – 47 760

≥ 40% hypercontractile contractions	24 (67)
≥ 50% hypercontractile contractions	14 (39)
≥ 80% hypercontractile contractions	5 (14)

*Basal mean LES pressure*	
Normal (10 – 45 mmHg)	28 (78)
Hypertonia (> 45 mmHg)	8 (22)

*LES relaxation*	
Normal median IRP (< 20 mmHg)	28 (78)
Elevated median IRP (≥ 20 mmHg)	8 (22)

Normal mean residual LES pressure (< 8 mmHg)	29 (81)
Elevated mean residual LES pressure (≥ 8mmHg)	7 (19)

DCI: distal contractile integral. LES: lower esophageal sphincter. IRP: integrated relaxation pressure.

**Table 4:** Complementary investigations in the patients with Jackhammer esophagus.

*Investigations*	*Normal*	*Abnormal*
(available results)		
*Esogastroduodenoscopy* (26)	18	(i) Hiatal hernias (3)
		(ii) Esophageal dilatation (2)
		(iii) LES hypertonia impression (2)
		(iv) Longitudinal striae (1)

*Esophageal biopsies* (13)	11	(i) Lymphocytic exocytosis (1)
		(ii) Esophagitis without eosinophilia (1)

*24 hours' pH-monitoring* (10)	5	(i) Pathological gastroesophageal reflux (4)
		(ii) Hypersensitive esophagus (1)

*Barium swallow* (8)	4	(i) Spastic esophageal contractions (3)
		(ii) Incomplete relaxation of the cricopharyngeal muscle (1)

## Data Availability

The demographic and clinical data used to support the findings of this retrospective observational study are available from the corresponding author upon request.
